# Personality of Wild Male Crested Macaques (*Macaca nigra*)

**DOI:** 10.1371/journal.pone.0069383

**Published:** 2013-08-05

**Authors:** Christof Neumann, Muhammad Agil, Anja Widdig, Antje Engelhardt

**Affiliations:** 1 Junior Research Group of Primate Sexual Selection, German Primate Center, Göttingen, Germany; 2 Courant Research Center “Evolution of Social Behavior”, Georg-August-Universität, Göttingen, Germany; 3 Junior Research Group of Primate Kin Selection, Max-Planck-Institute for Evolutionary Anthropology, Leipzig, Germany; 4 Institute of Biology, Faculty of Bioscience, Pharmacy and Psychology, University of Leipzig, Leipzig, Germany; 5 Faculty of Veterinary Medicine, Bogor Agricultural University, Bogor, Indonesia; Tulane University Medical School, United States of America

## Abstract

Animal personalities, i.e. consistent differences in behavior across time and/or context, have received increased attention of behavioral biologists over the last years. Recent research shows that personalities represent traits on which natural and sexual selection work and which can have substantial fitness consequences. The aim of this study is to establish the personality structure of crested macaque (*Macaca nigra*) males as foundation for future studies on its adaptive value. We collected behavioral data through focal animal sampling and additionally conducted two sets of playback experiments. Results of a factor analysis on the behavioral data revealed a four factor structure with components we labeled Anxiety, Sociability, Connectedness and Aggressiveness. Results from the experiments revealed an additional and independent Boldness factor but the absence of Neophilia. Overall, this structure resembles other macaque and animal species with the exception of Connectedness, which might be a consequence of the species' tolerant social style. Our results thus not only form the basis for future studies on the adaptive value of personality in crested macaques but also contribute an important data point for investigating the evolution of personality structure from a comparative perspective by refining, for example, which personality factors characterized the last common ancestor of hominids and macaques.

## Introduction

In recent years, the phenomenon that individuals in many, if not all, animal species differ from each other consistently in their behavior has received increasing attention from biologists and psychologists [1], [2]. If such individual differences are stable over time and/or across contexts they are referred to as animal personality or temperament [3]. Animal personalities are identified either by humans familiar with the study subjects via rating items in questionnaires (e.g. [4]–[6]) or through observational/experimental data (e.g. [7]–[9]). Both approaches usually subject primary data to factor analysis or related statistical methods, which allow identification of underlying dimensions that are then referred to as personality factors. Generally, both approaches lead to similar results when describing the personality structure (the combination of all present personality factors) of a given animal species (e.g., [10], but see [11], reviewed in [12]). Since personality can have profound effects on reproductive fitness, it is an important feature of animal biology that selective processes can work on [13]–[15].

Personalities of non-human primates (from here on: primates) are reasonably well studied (reviewed in [16]) due to the phylogenetic proximity of primates to humans and the complexity of the social systems exhibited by most primate species [17]. This growing body of data allows us to investigate the biological roots of human personality [1], [2], [18] and recently, a first formal attempt was made to describe the evolution of personality within the catarrhine primates ([4], see also [19]). Weiss and colleagues [4] hypothesize that the personality structure of humans is the evolutionary consequence of a series of changes along our phylogenetic history, with the human personality structure resembling more other hominid personality structures than those of more distantly related species such as rhesus macaques (*Macaca mulatta*). They further suggest that degree of sociality (i.e., differences in social organization and social relationships) has been the major selective pressure leading to the observed inter-specific differences in personality. This latter proposition however raises the question as to how personality structures differ in closely related species in which sociality may not be confounded by overall social organization (as is likely to be the case, for example, within the hominids).

In an alternative scenario, it has been suggested that the evolution of different types of sociality in different species is the result of differences in personality [20], [21]. For example, species in which individuals prefer close spatial proximity with conspecifics (an exemplified personality factor) may face increased feeding competition as compared to species in which individuals prefer greater spatial distance to conspecifics. This might then lead to more intense or more frequent aggressive behavior, which in turn may lead to the evolution of specific conflict management strategies not found in species that show lower levels of proximity [20]. This example illustrates how differences in personality could lead to changes in patterns of social behavior and shape social styles as sets of correlated behavioral patterns [21].

Among primates, the macaques (*Macaca*) represent a particularly well suited genus to look into the evolution of personality structures and its link to social behavior. On the one hand all of the approximately 20 species share some fundamental features of social organization such as living in temporally relatively stable multi-male/multi-female groups and male dispersal around sexual maturity [22]. On the other hand, macaques differ markedly in social style ([23], see also [24]–[26]), for example in regard to patterns of aggression and the degree of dominance asymmetries, conflict management strategies, and degree of kin bias in social interactions (e.g. [21], [26]–[28]). This particular diversity of social traits has led to the classification of macaque species into four grades of social styles ranging from so called despotic to tolerant species [23] and therefore represents an interesting model to study the link between sociality and personality.

Several macaque species have been studied in the context of personality. Our current knowledge about macaque personalities nevertheless comes foremost from studies on a single species, the despotic rhesus macaque (e.g. [4], [6], [29], [30]). In this species, at least three personality factors have been determined consistently across different studies: sociability (also labeled affiliation or not being solitary), aggressiveness (also labeled hostility), and fearfulness (also labeled excitability). These three factors seem to have deep phylogenetic roots beyond primates given that they appear not only in macaques [5], [31], but also in many other non-primate taxa (reviewed in [2]). Incidentally, they are thought to correspond to three dimensions of the dominating model of human personality – the Five Factor Model, i.e. Extraversion, Agreeableness, and Neuroticism [2], [32], [33]. Despite these possible similarities, there are also differences in personality structure between different macaque species and deviations from the human model. For instance, a distinct and separate personality factor Dominance [4], [19], [34], [35] has been described for hominids, but not for humans, and similar variation can be observed in the macaques: whereas in despotic rhesus macaques Dominance has been described as a distinct personality factor [4], it seems to be absent in the more tolerant Barbary macaques (*M. sylvanus*, [5]). Collectively, the available data suggest that personality structures of macaque species share some similarities but simultaneously show differences that are possibly linked to differences in species-specific social styles.

As illustrated within the macaques, this interplay of personalities and social styles is still poorly understood and certainly more data in all these domains are needed to form the basis for broader inter-species comparisons. An extensive body of data on macaque personality, social behavior and ecology already exists, that allows us to investigate this interaction within this interesting genus. Still, species at the tolerant end of the spectrum of social styles are underrepresented in all these respects and to the best of our knowledge, studies assessing personality structure in the most tolerant macaque species are completely missing.

The overall aim of this study is therefore to describe the personality structure of crested macaque males (*M. nigra*) as a foundation for future studies on its adaptive value. Following the macaque-typical pattern, crested macaques live in permanent multi-male/multi-female groups from which males disperse around reaching adulthood. Crested macaques have been classified into the very tolerant end of the macaque social style spectrum (c.f. [23]), based on the observation that, for example, social networks are diverse and that aggressive conflicts are frequently reconciled, and are of relatively low intensity with frequent occurrence of counter-aggression [21], [26], [27], [36], [37]. We therefore expect crested macaque personality to be more similar to personalities of tolerant species as compared to more despotic species. In particular we expect it to reflect the species-typical tolerant social style, which should manifest itself in the absence of a Dominance factor and the emphasis of factors in the social positive domain [5], [9]. In describing the crested macaque personality structure, we will enhance our knowledge on personality structures in tolerant primates as well as contribute to the clarification of the evolutionary history of personality within the macaques and primates including humans in general.

## Methods

### Ethics Statement

All research was conducted non-invasively on a wild population of crested macaques and in accordance with the Animal Behaviour Society's guidelines for the treatment of animals in behavioural research and teaching. In addition, we adhered to all relevant regulations of Indonesia and Germany. Permission to conduct the study in the Tangkoko-Batuangus Nature Reserve in Indonesia was granted by the Indonesian State Ministry of Research and Technology (RISTEK, permit 1189/FRP/SM/VI/2008), the Directorate General of Forest Protection and Nature Conservation (PHKA, permit SI.154/Set-3/2008) in Jakarta and the Department for the Conservation of Natural Resources (BKSDA, permit 58/SIMAKSI/BKSDA-SU/2009) in Manado. Since the study was non-invasive and approved by the local authorities in Indonesia, our institutions did not require approval by an ethics committee. *Macaca nigra* is classified as critically endangered [38] and our study did not affect the animals' welfare.

### Study subjects and site

Between March 2009 and May 2011, we studied 37 males of two wild, non-provisioned, groups of crested macaques, groups R1 and PB, living in the Tangkoko Nature Reserve, Sulawesi, Indonesia [27], [39]. The two groups comprised up to 85 individuals each, with 7–18 adult males present and are subject of research intermittently since the 1990's. All animals were completely habituated to human observers and adults were individually recognizable based on facial features and body markings, e.g., scars or broken limbs.

### Data collection

To assess personality, we used a combination of behavioral observations and experiments. First, we used *focal animal* and *scan sampling* [40] of 37 adult males (mean  = 66.1 h, range  = 0.6–130.0 h per male), and collected data on a range of specific behaviors and identities of other adults in spatial proximity of focal subjects. The selection of behavioral variables to include (Table 1) was designed to cover a broad field of social behavior and was based on a published account of the behavioral repertoire of crested macaques [41] and supplemented by variables suggested by a recent study on chimpanzee personality ([9], see also [10]). Focal protocols lasted 60 minutes during which continuous data on social and aggressive behavior and interaction partner identities were recorded. While following focal animals, these observational data were entered in handheld computers (Psion Workabout Pro G2) in real-time using spread-sheet software (PTab Spreadsheet v.3.0; Z4Soft). Additionally, we conducted scans at intervals of one, five, and thirty minutes to record general focal animal activity, identity of adult individuals in proximity, and whether the focal animal was outside vs. inside of the group, respectively (see Table 1). Data were collected by four observers and inter-observer reliability of the observed behaviors ranged between 0.75 and 1.00 as assessed by Pearson correlation coefficient and Cohen's kappa [42].

**Table 1:** Definitions of 22 behavioral variables.

Variable	Description
prop time spent active	Proportion of scan samples out of all scan samples not spent resting or self-grooming (at one minute interval)
prop time spent outside group	Proportion of scan samples in which animal was not in the center or periphery of the group; scans were taken twice per focal protocol: after 30 minutes and after 60 minutes
rate self-grooming	Hourly rate of self-grooming bouts (bouts were considered distinct if separated by > 2 seconds)
rate self-scratching	Hourly rate of self-scratching bouts (bouts were considered distinct if separated by > 2 seconds)
rate yawning	Hourly rate of yawns
rate status display	Hourly rate of loud call vocalization, a signal indicating dominance status [39]
prop time spent grooming	Proportion of scan samples out of all scan samples spent grooming other individuals (at one minute interval)
diversity grooming partners	Diversity index of adult females grooming was received from and given to; assessed from scan samples at one minute intervals
diversity grooming given	Diversity index of adult females grooming was given to; assessed from scan samples at one minute interval
number of female neighbors	Absolute number of adult female neighbors within five body lengths; assessed at scans every five minutes
diversity female neighbors (close)	Diversity index of adult females in close proximity (within one body length or in body contact); assessed during scans every five minutes
diversity female neighbors (far)	Diversity index of adult females in proximity (within five body lengths, but further than one body length); assessed during scans every five minutes
diversity male neighbors (close)	Diversity index of adult males in close proximity (within one body length or in body contact); assessed during scans every five minutes
diversity male neighbors (far)	Diversity index of adult males in proximity (within five body lengths, but further than one body length); assessed during scans every five minutes
rate approaching males	Hourly rate of approaching adult males within a range of five body lengths
rate approaching females	Hourly rate of approaching adult females within a range of five body lengths
rate affiliation towards males	Hourly rate of affiliative behavior (lip smack, mount, genital touch, friendly touch, play) directed at adult males
rate affiliation towards non-males	Hourly rate of affiliative behavior (lip smack, mount, genital touch, friendly touch, play) directed at individuals other than adult males
rate threats towards males	Hourly rate of threats directed at adult males
rate threats towards non-males	Hourly rate of threats directed at individuals other than adult males
rate aggression towards males	Hourly rate of overt aggression (bite, chase, hit) given to adult males
rate aggression towards non-males	Hourly rate of overt aggression (bite, chase, hit) given to individuals other than adult males

For more detailed description of behaviors and calculation of indices see [9], [41], [48].

Second, we used playback experiments on 18 of our focal males, to assess two possible personality factors that are hard to quantify by passive observations alone: boldness and neophilia [9], [43]. Boldness was assayed with the presentation of a dog bark bout (audio S1). Both crested macaque groups from time to time meet dogs from the nearby village in the forest and give alarm calls upon their sighting [Engelhardt et al., pers. obs.]. Neophilia was measured as the reaction to a donkey bray (unknown to test animals, audio S2). We conducted a total of 57 (n = 17 males) and 43 (n = 16 males) experimental trials in the dog and donkey condition, respectively. Males were presented with each of the stimuli repeatedly (up to 3 times), two consecutive trials of the same condition being separated by at least three weeks, and each male participating only once per day in an experiment. Stimuli were presented from a concealed DavidActive speaker (Visonik, Germany) connected to a Marantz PMD660 Flash-Disc recorder, placed 10–20 meters from the subject. The speaker was operated by an assistant hiding behind natural obstacles, for instance a tree trunk or buttress root, in such a way that neither the assistant nor the speaker was visible to the subject. Playbacks were only carried out when the subject sat calmly on the ground facing the direction in which the group was generally travelling. The speaker was placed in a 90° (±45°) angle relative to the subject's body orientation (see [44] for more details and an illustration of the experimental setup). The response to the playback stimulus was filmed with a digital video camera (Sony DCR-HC 90E) operated by the experimenter standing at a distance of about five meters from the subject (see video S1). In each experimental condition, we used the same recording as stimulus during all trials. We can therefore not rule out that subjects habituated to the repeated presentations of the same stimulus. Analytical methods, however, allow accounting for this possible bias in results (see methods on adjusted repeatabilities below).

### Data analysis

#### Behavioral data

We divided the overall data collection period into blocks of two months and calculated frequencies of behaviors, number of individuals in proximity and indices describing the diversity of individuals in proximity and grooming partners in each of these time blocks separately for each male (Table 1). There are two reasons for creating these time blocks. First, we wanted to assess temporal stability of our behavioral variables by calculating repeatabilities and repeated measurements are needed for each individual to obtain this measure [45], [46]. Second, group composition, particularly with respect to adult males, changed frequently [47]. Such an approach would lead to difficulties in the calculation of diversity indices, because in order to make these indices comparable they need to be standardized by accounting for maximum number of potential interaction partners [48].

An individual data point was included if the cumulative observation time for a given male and time block was at least six hours. If necessary, the raw behavioral variables were transformed (log, square root, arcsine) to achieve symmetric distributions. Variables were then standardized to a mean of zero and a standard deviation of one. Subsequently, all variables were tested for repeatability [45], [46]. We considered variables to be significant if the 95% confidence interval around their repeatability estimate did not include zero. Only significantly repeatable variables, i.e. variables that showed temporal stability, were subsequently subjected to factor analysis (see below). Repeatability estimates we present are well in the range of other studies on primates and non-primates (e.g., [9], [43], [49]–[51]). We tested the behavioral variables for group differences by means of Mann-Whitney U tests. One variable (number of female neighbors) differed significantly between the two study groups. Recalculating repeatability controlling for this group difference lowered the repeatability estimate of this variable, but did not change its statistical significance.

Since factor analysis requires independent data, and our data structure consisted of repeated measurements of individuals, we averaged values of the two-month time blocks to obtain single values for each male, thereby avoiding pseudo-replication. This procedure resulted in a data set comprising 30 males, with at least one two-month time block during which a male was observed for at least six hours.

The Kaiser-Meyer-Olkin measure of sampling adequacy (KMO  = 0.60) indicated that our data were suitable for factor analysis [52], even though overall sample size and case-variable ratio were small. We performed our analysis using the correlation matrix and a minimum residual solution. We decided to extract four factors based on visual inspection of a scree plot and eigenvalues [52]. We used an oblique (type “oblimin”) instead of an orthogonal rotation to allow for factors to be correlated [53]. We chose this approach because there is no a priori reason to assume that personality factors are independent of each other [52]. To gauge the relative importance of the behavioral variables to the extracted factors, we used factor loadings [52]. For interpretation, we considered variables that loaded saliently (absolute value ≥0.40) to contribute to a given factor [52]. If a variable loaded saliently on more than one factor we interpreted this variable as contributing to the factor on which it loaded with the highest absolute value (e.g., [10]).

#### Playbacks

Male responses to the playback stimuli from all experimental trials were coded from videotapes frame-by-frame by two coders, one being blind to study design and experimental condition. We discarded 19 experimental trials that were judged non-valid by both coders, due to technical problems, or subject distraction. Response variable was the time subjects oriented themselves towards the speaker in the first 10 seconds after the start of the stimulus presentation (hereafter: orientation duration) [44], [54]. We considered such a response to occur when the subject oriented its head towards or approached the speaker within an angle of about 22.5°. We considered stronger responses, i.e. longer orientation durations, to indicate bolder and more neophilic males. Both raters expressed high agreement in the orientation duration these responses had (Pearson correlation: r = 0.92, N = 81, p<0.001). Average duration values from both coders were used in subsequent analyses. We calculated adjusted repeatabilities (*R*_adj_, c.f. [45]) controlling for trial number within male subject and experimental condition to account for possible habituation effects. Since this algorithm does not permit confidence interval borders to be smaller than zero, we used p-values to determine statistical significance [45].

Finally, we computed correlations between the factors as well as between the factors and the responses to the two experiments. For this we extracted regression scores from the factor analysis for those males that were also subjects in the playback experiments, i.e. N = 17 (dog condition) and N = 16 (donkey condition). For the experimental data we used average male orientation durations (within each condition) for this calculation. We calculated Pearson correlation coefficients and since we had no specific hypotheses regarding possible relationships between personality factors, corrected the resulting p-values for multiple testing [55]. All analyses were conducted in R 2.15.0 [56] with the packages psych [57] and rptR [58].

## Results

### Behavioral data

The majority of the 22 behavioral variables were moderately repeatable, indicating that their expression was stable over time (Table 2). Five of the behavioral variables we considered were not repeatable, from which four reflected behavior towards other adult males: diversity of male neighbors (in close proximity) and the rates with which other males were approached, threatened and aggressed. In addition, rates of affiliation directed at individuals other than adult males, were not repeatable.

**Table 2:** Repeatabilities and confidence intervals of behavioral variables.

behavior	*R*	CI_l_	CI_u_
prop time spent active	0.33	0.16	0.51
prop time spent outside group	0.21	0.05	0.36
rate self-grooming	0.30	0.13	0.47
rate self-scratching	0.33	0.16	0.50
rate yawning	0.48	0.31	0.65
rate status display	0.70	0.57	0.83
prop time spent grooming	0.20	0.04	0.35
diversity grooming partners	0.16	0.02	0.31
diversity grooming given	0.26	0.10	0.42
number of female neighbors^a^	0.55	0.39	0.72
diversity female neighbors (close)	0.20	0.05	0.35
diversity female neighbors (far)	0.26	0.10	0.43
**diversity male neighbors (close)**	**0.10**	**−0.03**	**0.23**
diversity male neighbors (far)	0.23	0.07	0.39
**rate approaching males**	**0.09**	**−0.04**	**0.21**
rate approaching females	0.19	0.04	0.34
**rate affiliation towards non-males**	**0.12**	**−0.01**	**0.26**
rate affiliation towards males	0.16	0.02	0.31
rate threats towards non-males	0.26	0.09	0.42
**rate threats towards males**	**0.01**	**−0.09**	**0.10**
rate aggression towards non-males	0.14	0.00	0.28
**rate aggression towards males**	**−0.01**	**−0.10**	**0.08**

**Variables for which the confidence interval included zero (bold) were excluded from the subsequent factor analysis.**

a after controlling for group differences: *R* = 0.14, CI_l_  = 0.00, CI_u_  = 0.27.

The factor analysis based on the remaining 17 repeatable variables explained 62% of the total variance. All variables loaded on at least one of the four factors with an absolute value of >0.4 with six variables loading on two factors. The solution of loadings of variables onto the extracted factors after oblimin rotation is presented in Table 3.

**Table 3:** Loadings of the four extracted personality factors after oblimin rotation. Only loadings with absolute values ≥0.40 and communalities (*h* ^2^) are reported.

behavioral variable	anxiety^a^	connectedness	sociability	aggressiveness	*h* ^2^
prop time spent active	−0.89				0.88
rate self-grooming	0.45				0.34
rate self-scratching	0.90				0.84
rate status display	−0.41				0.24
rate approaching females	−0.74	(0.41)			0.81
prop time spent outside group	(0.48)	−0.49			0.74
diversity female neighbors (close)		0.84			0.88
diversity female neighbors (far)		0.77			0.61
diversity male neighbors (far)		0.44		(0.42)	0.49
diversity grooming given	(−0.46)	0.47			0.48
rate yawning		−0.64		(0.46)	0.61
prop time spent grooming			0.68		0.50
number of female neighbors	(0.48)		0.55		0.56
rate affiliation towards males			−0.73		0.55
diversity grooming partners			0.68		0.82
rate threats towards non-males				0.89	0.80
rate aggression towards non-males				0.64	0.43
**Eigenvalue**	3.52	2.90	2.26	1.90	
**Proportion Variance explained**	0.21	0.17	0.13	0.11	
**Cumulative Variance explained**	0.21	0.38	0.51	0.62	

**Values in brackets were not interpreted as belonging to this factor as they loaded higher on a different factor.**

Analysis based on correlation matrix (N = 30).

a loadings were reflected.

The first factor we extracted explained 21% of the variance with loadings of anxiety related behaviors (self-grooming, self-scratching). Additionally, males scoring higher on this factor were less active, gave less dominance displays (loud calls) and approached females more rarely. We labeled this factor *Anxiety* (Table 3).

The second factor explained 17% of variance and included variables that reflect diversity of male and female neighbors (diversity of close female neighbors, diversity of far female neighbors, diversity of far male neighbors) and the diversity of female grooming partners (female groomees). Additionally, this factor was related to smaller frequencies of yawning and spending more time in the core of the group. We labeled this factor *Connectedness* (Table 3).

The third factor accounted for 13% of variance with variables reflecting general social behavior, i.e. proportion of time spent grooming, diversity of grooming partners in general, and number of female neighbors. Interestingly, positive (affiliative) behavior directed at adult males loaded negatively on this factor. We named this factor *Sociability* (Table 3).

The fourth factor explained 11% of variance and reflected threat and overt aggression directed at individuals other than adult males. We labeled this factor *Aggressiveness* (Table 3).

### Playback experiments

Males reacted to the dog stimulus in all of the 39 trials. In the donkey condition, males reacted in 36 trials, while no reaction of males was visible on the video tapes in 6 trials. We found significant repeatability in response to the dog condition (*R*_adj_  = 0.55, 95% CI: 0.20–0.63, p = 0.002). We therefore considered the responses to this playback reflecting *Boldness*. In contrast, we did not find responses to the donkey condition to be repeatable (*R*_adj_  = 0.05, 95% CI: 0.00–0.27, p = 0.099) and therefore consider *Neophilia* to be absent.

### Relationships between the factors

After controlling for multiple testing, we did not find any significant correlation between any pair of the four observationally assessed factors and the boldness factor (Pearson correlation coefficients, mean  = −0.05, range  = −0.39–0.37, p value range: 0.047–0.940, ten pairwise comparisons, Table 4). In addition, we found no significant relationship between any of these five factors and orientation duration in response to the donkey playback (Pearson correlation coefficients, mean  = −0.02, range  = −0.39–0.32, p value range: 0.131–0.877, five pairwise comparisons, Table 4).

**Table 4:** Pearson correlation coefficients between factors and the responses to the donkey playback.

	connectedness	sociability	aggressiveness	boldness	donkey playback
**anxiety**	−0.19	−0.17	0.09	−0.39	−0.15
**connectedness**		0.37	−0.07	−0.13	−0.04
**sociability**			0.01	0.35	0.14
**aggressiveness**				−0.33	−0.39
**boldness**					0.32

N  =  17 for correlations with boldness, N = 16 for correlations with responses to donkey playback, and N = 15 for correlation between boldness and responses to donkey playback.

## Discussion

Our results on observational and experimental data suggest that crested macaque personality comprises five distinct and unrelated factors: Anxiety, Connectedness, Sociability, Aggressiveness and Boldness (summarized in Table 5). This structure is generally similar to the structures observed in other macaque species (e.g., [4], [5], [31]). In addition, the structure our data suggest is characterized by the presence of two distinct factors that reflect socio-positive behavior (Connectedness and Sociability). Most notably, we identified a factor, Connectedness, which, to our knowledge, has not been described in studies of primate personalities before, and which covers aspects of social network diversity, a feature that might be of particular importance in tolerant as opposed to despotic primate species.

**Table 5:** Summary of personality factors for crested macaque males.

Personality factor	Description
**Anxiety**	High rates of self-directed behavior, reluctance to approach females
**Connectedness**	Diverse neighbor and grooming network, spatial position in the core of the group
**Sociability**	High rate of grooming, high number of female neighbors, diverse grooming network
**Aggressiveness**	High rates of threats and aggression
**Boldness**	Reacts strong towards threatening situation

**Descriptions refer to animals scoring high on the respective factor.**

Our study is focused on adult males because ultimately we are interested in the fitness consequences of personality among males. Given this, we cannot exclude the possibility that our description of the personality structure of crested macaques is incomplete. However, to our knowledge no empirical study so far has shown sex-specific differences in personality structure within a species.

Anxiety, Aggressiveness and Sociability in our study matched factors that have been described in previous studies of macaque personalities [4], [5], [31] and are widespread among other primate and non-primate species (reviewed in [2], [16]). Anxiety is commonly used to describe general unease and distress [4]. That the factor we name Anxiety reflects the individual degree of unease and distress in our study species is evident through the loading of self-directed behavior onto this factor, which is a behavioral manifestation of physiological stress levels [59]. In our study, males scoring high on Anxiety approached females less frequent than less anxious males. Anxiety might therefore reflect the reluctance or willingness of males to approach females, possibly mediated through general unease whilst in female proximity (see also [60]). In addition, our measure of general activity also loaded on the Anxiety factor. Similarly, in Barbary macaques, questionnaire items, i.e. adjectives to be rated by human observers, typically describing distress (e.g., *tense*, *irritable*, *excitable*) loaded on one single factor alongside items that describe general activity (*active, lazy*). Consequently, this factor has been labeled Activity/Excitability in this study [5]. We suggest, however, that Anxiety might in fact be the more fitting label for crested and possibly also Barbary macaques since it captures more of an intrinsic feature as compared to Activity which may well be constrained by external factors, such as the environment. For example, Barbary macaques are more active (i.e. they rest less) when their home-range is covered with snow [61]. Given that Barbary macaques are also classified as relatively tolerant, this combination of anxiety and activity represented in only one personality factor might constitute a general feature of the more tolerant macaque species. In contrast, in despotic rhesus macaques both factors are distinct ([4], see also [9] for this pattern in chimpanzees, *Pan troglodytes*).

In crested macaque males, the personality factor Aggressiveness covers threat behavior and overt aggression directed at females, sub-adults, juveniles and infants. Interestingly, aggressive behavior directed at adult male group members was not part of this factor. The reason for this was that rates of aggression towards adult males were not stable over time and hence not included into our factor analysis. As previously mentioned, we observed frequent changes in composition as well as in dominance relationships among adult males in our groups, whereas among adult females, group composition and dominance hierarchy remained much more stable over the course of our study [27], [47]. It could be argued that temporal stability or instability in behavior is the consequence of a stable or instable social environment, and consequently, stable behavioral patterns may not reflect an intrinsic property (i.e. personality), but rather are the result of external constraints [9], [62]. Therefore, the lack of repeatability in our study of aggression towards other males may have been a consequence of these dynamics. Indeed, having a stereotypic aggression rate towards other males regardless of the dynamics among adult males might be mal-adaptive in the sense that this might lead to costs imposed by overly frequent and possibly injuring aggression depending on how many males co-reside in the group.

Aggressiveness has also been found in other primate (including macaques) and non-primate species, but it is frequently labeled Confidence or Dominance in studies based on observers' ratings such as *aggressive, bullying, dominant, submissive and confident* ([4], [5], [30], but see [31]). It often correlates with behaviorally assessed (agonistic) dominance ranks [5], [10], [29], [63], [64]. In studies of animal behavior, dominance is however considered a dynamic individual property that changes over time due to external events (e.g., challenges by other individuals or migration) and which, in the strictest sense, is the property of a single individual within a dyad where the other individual is subordinate at a given time [65]). Given the possible confusion with the term *dominance* as used in behavioral biology ([2], [65]; see Capitanio [11] for an illustrative example how such confusion between personality Dominance and behavioral dominance might easily arise) we suggest that the label of the Dominance facet of primate personality should be reevaluated. For rhesus macaques, for example, based on the items that describe it (see above) the label Dominance could, in alignment with our study, be replaced by Aggressiveness. Such more consistent labeling of personality factors would facilitate inter-specific comparisons.

The final personality factor that crested macaques share with other macaques is Sociability. This factor covers behavior that seems essential for building and maintaining social relationships with female group members, particularly the amount of grooming, the diversity of grooming partners and the number of female neighbors. Sociability as defined in our study, matches the Friendliness and Sociability components of other macaque species' personality structures [4], [5], [30], [31], in which it is associated with rating items like *sociable, gentle* and *friendly* [4], [5].

In addition to the three above mentioned personality factors that appear to generally occur in macaques, we found a further personality factor in crested macaques, Connectedness. Interestingly, this factor in addition to Sociability also covers behavior in the socio-positive domain. It describes the diversity of females which are groomed by a specific male, the diversity of adult individuals in proximity to him and the time males spend in the core of their social group. Connectedness has no obvious homologue in other macaque species. However, given the overall socio-positive notion of this factor we speculate that in rhesus and Barbary macaques it may be part of the Friendliness factor. Consequently, whereas Friendliness (or Sociability) constitutes a single factor in some species, we find that in crested macaques, socio-positive behavior is reflected in two distinct personality factors, Sociability and Connectedness.

Finding such an additional socio-positive dimension might be a consequence of the complex social network that individuals of tolerant macaque species, such as crested macaques, have as compared to individuals of more despotic species [23], [31]. This wide network connecting also not related individuals shows that relationships between group members are far less constrained than in despotic species where relationships are maintained predominantly within fixed kin networks (e.g., [28], [66]). Two possible ways in which scoring high on such a personality factor might thus be biologically adaptive (fitness increase) are through a more diverse network of allies that could provide support in agonistic conflicts or through increased attractiveness to the opposite sex (e.g., [67]–[70]). Further studies will be necessary to determine whether Connectedness indeed has fitness benefits and whether it is a general feature of socially tolerant primates such as crested macaques.

The final personality factor we identified in crested macaque males is Boldness as evidenced by repeatable reactions towards the auditory presentation of a threating stimulus. Boldness has rarely been studied in primates in general [16]. Such a factor might however evolve under the selective pressure of predation, particularly in species in which predators are mobbed upon detection as is the case for crested macaques on sight of reticulated pythons (*Python reticulatus*), their presumed primary predator [44]. Scoring high in Boldness could under these circumstances not only help to roust the predator, but may also be used as a potentially costly signal of social status or as a means of attractiveness to potential mating partners. In this sense, we cannot rule out the possibility that the composition of the audience present during the playback trials influenced males' reactions to the stimulus. Intuitively, crested macaque Boldness appears to be similar to the Confidence factor found in other macaques. This notion is however rather speculative and based on rating items used in questionnaire studies, such as *fearful* or *timid* [4], [5]. At the same time, since we also consider our Aggressiveness factor as equivalent to rhesus and Barbary Confidence, we would have to expect a salient positive correlation between crested macaque Boldness and Aggressiveness, which was absent. For now, we hypothesize that Boldness makes up a unique factor in crested macaques, but cannot rule out that this is due to the different approaches with which we determined Boldness and Aggressiveness. Given the prevalence of Boldness in non-primate animals in general [1], [3] and its hypothetical absence in primates ([16], but see [43]), future studies of primate personality should incorporate either items like *bold* into their questionnaires or try to assess Boldness experimentally [43]. This should clarify in how far Boldness constitutes an independent factor of primate personality, or is just a facet of some other factor, such as Aggressiveness or Confidence.

In agreement with most studies of macaques, we found no evidence for the existence of a distinct Neophilia dimension (but see Openness with the items *curious* and *inventive* in rhesus macaques, [4]). Neither did we observe any significant correlation between the response to our novelty experiments and any of the other five personality factors. This absence is somewhat surprising, since a Neophilia/Exploration factor (or the human equivalent Openness) is present in many animal species including primates ([2], [3], [71], see also [72], [73]). However, the actual correlation coefficients of the playback response with Aggressiveness and Boldness were modest, but not negligible (−0.39 and 0.32), suggesting that the absence of significant relationships in our study might potentially have been a problem of statistical power or inappropriate stimulus choice [43]. In line with this possible overlap with other factors, studies in other macaque species found rating items that describe Neophilia such as *curious* or *exploratory* load on diverse factors such as Sociability [30], [63], Friendliness [4] or Activity/Excitability [5], [6]. An alternative explanation for the absence of Neophilia may be related to the modality in which we presented the stimulus. Macaques, as many primates, are vision-dominant, suggesting that an auditory stimulus may be less likely to elicit consistent responses in our experimental setup. More studies are certainly needed to confirm or reject the absence of Neophilia in macaques.

Based on our results, some tentative comparisons can also be made between macaque and human personality in order to investigate the evolutionary roots of the current human personality structure as posited in the Five Factor Model [4], [32]. The combination of the two socio-positive factors we found in crested macaques (Connectedness and Sociability) appear to match human Extraversion, whereas Aggressiveness may reflect the human Agreeableness vs. antagonism axis [2]. Anxiety and, to a lesser degree, Boldness fit the descriptors of human Neuroticism [2]. In contrast, human Conscientiousness and Openness appear not to be reflected in crested macaque personality as distinct factors. Although crested macaque personality factors do not match perfectly with those of humans, our results shed important light onto the evolution of personality structures within the primate taxon.

Our attempt to directly link crested macaque with human personality structure is however a simplification of the hypothetical evolutionary scenario proposed to explain the evolution of human personality structure from a macaque-like ancestor [4]. Here we will use two examples to illustrate how incorporating data from additional species may help to formulate alternative hypotheses to explain the evolutionary history of personality structures. In their personality phylogeny using rhesus macaques as an outgroup to hominids, Weiss and colleagues [4] suggest that Anxiety and Activity are two distinct ancestral factors. Our data and recent work on Barbary macaques, however, allow an alternative scenario. In both Barbary and crested macaques, a single factor describes a combination of Anxiety and Activity (labeled Anxiety in crested macaques (see above) and Activity/Excitability in Barbary macaques [5]). Given that Barbary macaques represent the sister taxon to all other extant macaque species [74], [75], it may thus be that such a broader, singular Anxiety/Activity factor is the actual ancestral state. In addition, the presence of a single Sociability factor in Barbary and rhesus macaques may also represent the ancestral macaque state, whereas the occurrence of the two distinct factors we identified in crested macaques (Sociability and Connectedness) most likely is a derived feature. We therefore speculate that a hypothetical ancestor to macaques (and hominids) was characterized by a single Anxiety/Activity factor and a single Sociability factor, similar to present-day Barbary macaques [5].

Based on this suggested proto-macaque, we can formulate alternative hypotheses of how personality factors evolved in higher lineages. For example, according to a recently proposed scenario, the hominid Extraversion factor evolved from a combination of a “pure” Activity factor and a Sociability factor [4]. Our results, however, indicate the possibility that such a single Activity factor was not the ancestral state of catarrhine primates. Hence, in order to evolve Extraversion in the hominid lineage (c.f. [4]), a separation of our suggested ancestral Anxiety/Activity factor into two distinct factors needs to occur, after the phylogenetic split of hominids and macaques. With the currently available data, both scenarios are equally parsimonious and only future studies that contribute more data will help to fully understand the evolution of primate personality structures.

Whereas a thorough treatment of the possible evolution of primate personalities is well beyond the scope of this paper, we find overall support for the hypothesis of Weiss and colleagues [4] in that crested macaque personality structure resembles more other macaque personality structures than hominid personalities. The comparative approach is thus surely a promising one and the more data points we acquire the clearer the overall picture of personality evolution will become. In addition, more and more data are being generated concerning neurobiological and endocrinological mechanisms underlying personality variation (e.g., [76]–[79]). By integrating these approaches with ethological data and with the recent progress in behavior genetics (e.g., [79], [80]), new avenues will open to study the evolutionary paths and selective pressures leading to the striking variation in personality structures we observe across the animal kingdom.

## Supporting Information

Audio file S1 **Audio recording of the dog bark used as stimulus in the playback experiments.**
(WAV)

Audio file S2 **Audio recording of the donkey bray used as stimulus in the playback experiments.**
(WAV)

Video S1 **Example of an experimental trial in the dog condition.** The speaker is placed left, from the viewer's perspective. Towards the end of the clip, an alarm call is audible given by an individual not visible in the frame.
(MOV)
